# Impact of Heat Stress on Poultry Production

**DOI:** 10.3390/ani3020356

**Published:** 2013-04-24

**Authors:** Lucas J. Lara, Marcos H. Rostagno

**Affiliations:** 1 Department of Animal Sciences, Purdue University, 915 West State Street, West Lafayette, IN 47907, USA; E-Mail: lucasjanuzzi@gmail.com; 2 Livestock Behavior Research Unit, USDA-ARS, 125 South Russell Street, West Lafayette, IN 47907, USA

**Keywords:** heat stress, poultry, broilers, laying hens, welfare, production, food safety

## Abstract

**Simple Summary:**

Due to the common occurrence of environmental stressors worldwide, many studies have investigated the detrimental effects of heat stress on poultry production. It has been shown that heat stress negatively affects the welfare and productivity of broilers and laying hens. However, further research is still needed to improve the knowledge of basic mechanisms associated to the negative effects of heat stress in poultry, as well as to develop effective interventions.

**Abstract:**

Understanding and controlling environmental conditions is crucial to successful poultry production and welfare. Heat stress is one of the most important environmental stressors challenging poultry production worldwide. The detrimental effects of heat stress on broilers and laying hens range from reduced growth and egg production to decreased poultry and egg quality and safety. Moreover, the negative impact of heat stress on poultry welfare has recently attracted increasing public awareness and concern. Much information has been published on the effects of heat stress on productivity and immune response in poultry. However, our knowledge of basic mechanisms associated to the reported effects, as well as related to poultry behavior and welfare under heat stress conditions is in fact scarce. Intervention strategies to deal with heat stress conditions have been the focus of many published studies. Nevertheless, effectiveness of most of the interventions has been variable or inconsistent. This review focuses on the scientific evidence available on the importance and impact of heat stress in poultry production, with emphasis on broilers and laying hens.

## 1. Introduction

Stress, a response to adverse stimuli, is difficult to define and understand because of its nebulous perception. According to Selye [1], “stress is the nonspecific response of the body to any demand”, whereas stressor can be defined as “an agent that produces stress at any time”. Therefore, stress represents the reaction of the animal organism (*i.e.*, a biological response) to stimuli that disturb its normal physiological equilibrium or homeostasis.

Heat stress results from a negative balance between the net amount of energy flowing from the animal’s body to its surrounding environment and the amount of heat energy produced by the animal. This imbalance may be caused by variations of a combination of environmental factors (e.g., sunlight, thermal irradiation, and air temperature, humidity and movement), and characteristics of the animal (e.g., species, metabolism rate, and thermoregulatory mechanisms). Environmental stressors, such as heat stress, are particularly detrimental to animal agriculture [2,3,4]. The issue of environmental stress has quickly become a great point of interest in animal agriculture, particularly due to public awareness and concerns.

The importance of animal responses to environmental challenges applies to all species. However, poultry seems to be particularly sensitive to temperature-associated environmental challenges, especially heat stress. It has been suggested that modern poultry genotypes produce more body heat, due to their greater metabolic activity [5,6]. Understanding and controlling environmental conditions is crucial to successful poultry production and welfare. Therefore, the objective of this review is to compile the current knowledge and evidence available in the scientific (peer-reviewed) literature examining what is known about the importance and impact of heat stress in poultry production, focusing on broilers and laying hens.

## 2. Behavioral and Physiological Effects of Heat Stress

Under high temperature conditions, birds alter their behavior and physiological homeostasis seeking thermoregulation, thereby decreasing body temperature. In general, different types of birds react similarly to heat stress, expressing some individual variation in intensity and duration of their responses. A recent study [7] showed that birds subjected to heat stress conditions spend less time feeding, more time drinking and panting, as well as more time with their wings elevated, less time moving or walking, and more time resting.

Animals utilize multiple ways for maintaining thermoregulation and homeostasis when subjected to high environmental temperatures, including increasing radiant, convective and evaporative heat loss by vasodilatation and perspiration [8]. Birds have an additional mechanism to promote heat exchange between their body and the environment, which are the air sacs. Air sacs are very useful during panting, as they promote air circulation on surfaces contributing to increase gas exchanges with the air, and consequently, the evaporative loss of heat [9]. However, it is worth noting that increased panting under heat stress conditions leads to increased carbon dioxide levels and higher blood pH (*i.e.*, alkalosis), which in turn hampers blood bicarbonate availability for egg shell mineralization and induces increased organic acid availability, also decreasing free calcium levels in the blood. This process is very important in breeders and laying hens, as it affects egg shell quality [10]. However, although many studies have attempted to characterize the physiological mechanisms associated to the egg quality decrease in heat stressed birds, there is no definitive knowledge, and several potential pathways are still under investigation, including changes of reproductive hormones levels and of intestinal calcium uptake [11,12]. Heat stress can affect the reproductive function of poultry in different ways. In females, heat stress can disrupt the normal status of reproductive hormones at the hypothalamus, and at the ovary, leading to reduced systemic levels and functions [13,14,15,16]. Also, negative effects caused by heat stress in males have been shown in different studies. Semen volume, sperm concentration, number of live sperm cells and motility decreased when males were subjected to heat stress [17,18,19].

High environmental temperatures alter the activity of the neuroendocrine system of poultry, resulting in activation of the hypothalamic-pituitary-adrenal (HPA) axis, and elevated plasma corticosterone concentrations [20,21,22,23]. Body temperature and metabolic activity are regulated by the thyroid hormones, triiodothyronine (T3) and thyroxine (T4), and their balance. Previous studies report that T3 concentrations consistently decrease in high temperature conditions [7,11,21,24,25], whereas results of heat-mediated alterations on T4 concentrations are inconsistent with studies reporting decrease [26], increase [11,27], or no alteration [7,28]. Due to the involvement of the thyroid during the onset of puberty and reproductive function in birds, a disruption of thyroid activity by heat stress would be expected to have an effect on reproductive performance of the hens [16]. Moreover, findings reported by Geraert *et al.* [24] indicate that endocrinological changes caused by chronic heat stress in broilers stimulate lipid accumulation through increased *de novo* lipogenesis, reduced lipolysis, and enhanced amino acid catabolism.

In summary, heat stress impairs overall poultry and egg production by modifying the bird’s neuronedocrine profile both by decreased feed intake and by activation of the HPA axis. In general, birds react similarly to heat stress, but express individual variation of intensity and duration of responses, which may also be affected by intensity and duration of the heat stress event. Another potential cause of variations resides in the fact that heat stress is often times not experienced in isolation, being usually accompanied by other stressors, such as limited housing space and insufficient ventilation, as well as social interactions and previous experiences, which have been shown to affect the individual’s stress response [29,30]. Additionally, increasing evidence indicates that much of the variation in response to heat stress is apparently genetically-based [7,31,32]. However, this area still requires further study to increase the currently limited knowledge available. In general, it needs to be kept in mind that animal welfare is a multifactorial concept, based on freedom from disease, ability to perform specific behaviors and to cope with social and environmental conditions [33].

## 3. Effect of Heat Stress on the Immune Response

Many studies have been conducted to elucidate how stress affects the immune response in animals. Modulation of the immune response by the central nervous system (CNS) is mediated by a complex network that operates bi-directionally between the nervous, endocrine and immune systems. The hypothalamic–pituitary–adrenal (HPA) and the sympathetic–adrenal medullar (SAM) axes constitute the main pathways through which the immune response can be altered. It has been shown that lymphocytes, monocytes or macrophages, and granulocytes exhibit receptors for many neuroendocrine products of the HPA and SAM axes, such as cortisol and catecholamines, which can affect cellular trafficking, proliferation, cytokine secretion, antibody production and cytolytic activity. This topic has been the subject of several extensive reviews [34,35,36,37]. However, knowledge continues to be generated, providing increasing insights on the interplay among the nervous, endocrine and immune systems.

In poultry, several studies have investigated the effects of heat stress on the immune response in recent years. In general, all studies show an immunosuppressing effect of heat stress on broilers and laying hens, although using different measurements. For instance, lower relative weights of thymus and spleen has been found in laying hens subjected to heat stress [38]; reduced lymphoid organ weights have also been reported in broilers under heat stress conditions [22,39,40]. Additionally, Felver-Gant *et al.* [32] observed reduced liver weights in laying hens subjected to chronic heat stress conditions. Bartlett and Smith [41] observed that broilers subjected to heat stress had lower levels of total circulating antibodies, as well as lower specific IgM and IgG levels, both during primary and secondary humoral responses. Moreover, they observed significantly reduced thymus, bursa, spleen, and liver weights. Aengwanich [42] also demonstrated the occurrence of reduced bursa weight in broilers subjected to heat stress, as well as decreased numbers of lymphocytes in the cortex and medulla areas of the bursa.

While reduced systemic humoral immune response has been reported [43], fewer intraepithelial lymphocytes and IgA-secreting cells in the intestinal tract of laying hens under heat stress have also been observed [44]. Others [39,40] have also reported reduced antibody response, as well as reduced phagocytic ability of macrophages, in broilers under heat stress. Moreover, reduced macrophages performing phagocytosis, as well as reduced macrophage basal and induced oxidative burst were observed in heat-stressed broilers [22,23]. Recent studies have also demonstrated that heat stress can alter levels of circulating cells. It has been shown that heat stress causes an increase in heterophil:lymphocyte ratio, due to reduced numbers of circulating lymphocytes and higher numbers of heterophils [32,45].

Under environmental stressful conditions, as the bird’s body attempts to maintain its thermal homeostasis, increased levels of reactive oxygen species (ROS) occur. As a consequence, the body enters a stage of oxidative stress, and starts producing and releasing heat shock proteins (HSP) to try and protect itself from the deleterious cellular effects of ROS [46]. In fact, higher concentrations of HSP70 were found in broilers and laying hens exposed to heat stress [32,47].

## 4. Impact of Heat Stress on Poultry Production

Many studies have been published about the effects of heat stress on the efficiency of broiler production. As previously seen, exposure of birds to high environmental temperature generates behavioral, physiological and immunological responses, which impose detrimental consequences to their productivity. Heat stress results in estimated total annual economic loss to the U.S. livestock production industry of $1.69 to $2.36 billion; from this total, $128 to $165 million occurs in the poultry industry [48].

In a recent study [49], broilers subjected to chronic heat stress had significantly reduced feed intake (−16.4%), lower body weight (−32.6%), and higher feed conversion ratio (+25.6%) at 42 days of age. Many additional studies have shown impaired growth performance in broilers subjected to heat stress [6,38,40,50,51]. However, even though the detrimental effects of heat stress in broilers seem to be very consistent, it is important to consider that stocking density has a major role as a potential compounding factor, both from the standpoint of productivity as well as welfare [52].

It has been reported that chronic heat exposure negatively affects fat deposition and meat quality in broilers, in a breed-dependent manner [53]. In fact, recent studies demonstrated that heat stress is associated with depression of meat chemical composition and quality in broilers [54,55]. Another recent study [56] demonstrated that chronic heat stress decreased the proportion of breast muscle, while increasing the proportion of thigh muscle in broilers. Moreover, the study also showed that protein content was lower and fat deposition higher in birds subjected to heat stress.

Broilers may be exposed to a variety of stressors during transport from the production farms to the processing facilities, including thermal challenges of the transport microenvironment, acceleration, vibration, motion, impacts, fasting, withdrawal of water, social disruption, and noise [57,58]. As part of this complex combination of factors, thermal stress, in particular heat stress, plays a major role. The confined conditions within the transport containers reduce the effectiveness of the bird’s behavioral and physiological thermoregulatory mechanisms [58]. Consequently, the adverse effects of these factors and their combinations range from mild discomfort to death. In fact, heat stress during transport has been associated with higher mortality rate, decreased meat quality, and reduced welfare status [57]. In a study conducted during the course of 3 years, Warriss *et al.* [58] demonstrated a seasonal impact with peak mortality rates occurring in the summer months. Moreover, the study showed a progressive, marked increase in broiler mortality as the environmental temperature increased. In a study to determine the factors influencing bruises and mortality of broilers at harvest [59], percentage of bruises was associated with season, moment of transport, and ambient temperature; the same factors were also associated with increased mortality, in addition to body weight and stocking density, transport and lairage time. Interestingly, it has also been reported that death in transit between production farms and processing facilities is associated with bird size (*i.e.*, larger birds = higher mortality risk) [60]. It is undisputable that the welfare of broiler production is becoming an increasing public concern in relation to both the production stage per se, but also to the harvest process. It is evident that not enough attention has been given to this area, and therefore, further research is critically needed.

Productivity of laying hens flocks may also be affected by a multitude of factors, including environmental stress (such as heat stress), which is probably one of the most commonly occurring challenges in many production systems around of the world. Decreased feed intake is very likely the starting point of most detrimental effects of heat stress on production, leading to decreased body weight, feed efficiency, egg production and quality [44,61]. However, in addition to decreased feed intake, it has been shown that heat stress leads to reduced dietary digestibility, and decreased plasma protein and calcium levels [62,63,64].

In a recent study [44], a 12-day heat stress period caused a daily feed intake reduction of 28.58 g/bird, resulting in a 28.8% decrease in egg production. Star *et al.* [65] reported a reduction of 31.6% in feed conversion, 36.4% in egg production, and 3.41% in egg weight in laying hens subjected to heat stress. In another study [66], heat stress caused decreased production performance, as well as reduced eggshell thickness, and increased egg breakage. Additionally, heat stress has been shown to cause a significant reduction of egg weight (−3.24%), egg shell thickness (−1.2%), eggshell weight (−9.93%), and eggshell percent (−0.66%) [12]. Corroborating these reports, Mack *et al.* [7] also observed decreased egg production, egg weight and egg shell thickness in laying hens subjected to heat stress. An interesting series of experiments [67] demonstrated the increasing detrimental effect that chronic heat stress has on egg production. In these experiments, a reduction of 13.2%, 26.4% and 57% occurred in egg production in laying hens subjected to heat stress during 8–14 days, 30–42 days and 43–56 days, respectively. In another study [61], a marked decrease in egg production (28.8%), feed intake (34.7%) and body weight (19.3%) was also observed in laying hens subjected to chronic heat stress, during a 5-week period.

Although much variation of effects is observed between many of the studies published, the consistent finding of significant impacts of heat stress on egg production and quality is noteworthy. The variability of the effects reported may be easily explained by the use of birds of different age or genetic background, as well as due to variable intensity and duration of the heat stress treatments applied.

## 5. Can Heat Stress Impact Food Safety?

Heat stress during the growth period of broilers has been associated with undesirable meat characteristics and quality loss [53,56,68]. Additionally, transportation of broilers from farms to processing facilities under high temperature conditions have also been shown to cause meat quality losses [69,70,71]. In laying hens, heat stress has been shown to negatively affect egg production and quality [43,61]. More recently, food safety has become a major issue to the poultry and egg production industry worldwide. In fact, food safety is increasingly being considered an important part of the modern food quality concept.

Colonization of birds by foodborne pathogens, such as *Salmonella* and *Campylobacter*, and their subsequent dissemination along the human food chain are a major public health and economic concern in poultry and egg production. In fact, consumption and handling of undercooked poultry products constitutes one of the most commonly implicated sources of foodborne illness [72,73,74,75].

There is increasing evidence to demonstrate that stress can have a significant deleterious effect on food safety through a variety of potential mechanisms. However, while there is evidence linking stress with pathogen carriage and shedding in farm animals, the mechanisms underlying this effect have not been fully elucidated [76,77,78]. Environmental stress has been shown to be a factor that can lead to colonization of farm animals by pathogens, increased fecal shedding and horizontal transmission, and consequently, increased contamination risk of animal products [77,78,79]. For a long time, these aspects of animal infections have been attributed to effects of stress-associated hormones and mediators on the immune system (mostly, due to immunosuppression). However, in recent years, a new perspective has been proposed, based on the direct effect of stress-associated hormones and mediators on bacterial pathogens, known as “microbial endocrinology” [80]. Many recent studies have demonstrated that bacteria, such as *Salmonella* and *Campylobacter*, are capable of exploiting the neuroendocrine alterations due to the stress response in the host to promote growth and pathogenicity [79,81]. Therefore, it is of great importance to be aware that environmental stresses, such as heat stress, can potentially alter the host-pathogen interaction.

The gastrointestinal tract is particularly responsive to stressors, which can cause a variety of changes, including alteration of the protective microbiota as well as decreased integrity of the intestinal epithelium [82,83]. Also, as previously discussed, there is considerable evidence to indicate that response and coping with environmental stressors can modify biological defense systems, such as antibody and cell-mediated immune responses, thereby increasing susceptibility to pathogens. The intestinal tract of poultry harbors a complex and dynamic microbial ecosystem (or microbiome), which may be affected by a variety of factors [84]. Very little has been published on the effects of environmental stressors (particularly, heat stress) on the intestinal microbial ecosystem of poultry. However, studies have been reported demonstrating that heat stress affects the microbial composition as well as the concentration of short-chain fatty acids in the rumen [85,86], which is a much more complex microbial system in comparison to the poultry intestinal microbiome. Therefore, it is reasonable to assume that heat stress would also affect the intestinal microbial populations of poultry. However, this knowledge gap still needs to be better explored and understood. For instance, heat stress has been shown to cause increased intestinal permeability in broilers [20]. Altered morphology, as well as changes in the microbial community structure in the intestinal tract of broilers subjected to heat stress have been reported [87]. Moreover, using an *ex vivo* approach, the same study [87] showed that mucosal attachment of *Salmonella* Enteritidis increased when tissues originated from heat-stressed birds. Corroborating the intestinal morphology alterations observed in the previous study [87], more studies [43,44] also observed morphological alterations in the intestinal tract of laying hens subjected to heat stress, consisting of decreased villus height and ratio of villus height to crypt depth.

Oxidative stress is the starting point of the intestinal permeability dysfunctional process. Under heat stress conditions, increased concentrations of reactive oxygen species (ROS) occur leading to increased intestinal permeability, which in turn facilitates the translocation of bacteria from the intestinal tract. In fact, increased inflammation and translocation of *Salmonella* Enteritidis in broilers subjected to heat stress has been reported [22,23], resulting in increased levels of the pathogen in spleen samples.

It is reasonable to speculate that high environmental temperature would not only affect the bacterial levels in the feces of birds, but also the duration and level of contamination in the environment where feces are deposited, potentially leading to increased dissemination. However, heat stress did not result in higher levels or longer survival of *Salmonella* shed in feces in a small study [88]. Nevertheless, several epidemiological studies have reported seasonal effects on the occurrence of *Salmonella* and *Campylobacter* in flocks of broilers and laying hens, as well as in retail poultry products [89,90,91,92,93]. Therefore, this area represents a critical gap of knowledge that needs to be filled, due to its wide implications to our understanding of the ecology and epidemiology of pathogens in poultry flocks under high temperature or heat stress conditions.

## 6. Conclusion and Final Considerations

Heat stress is one of the most important environmental stressors challenging poultry production worldwide. The negative effects of heat stress on broilers and laying hens range from reduced growth and egg production to decreased poultry and egg quality and safety. However, a major concern should be the negative impact of heat stress on poultry welfare. As presented in this review, much information has been published on the effects of heat stress on productivity and immune response in poultry (broilers and laying hens). However, our understanding of basic mechanisms associated to the reported effects, as well as related to behavior and welfare of the birds under heat stress conditions are in fact scarce.

Finally, it is important to mention that intervention strategies to deal with heat stress conditions have been the focus of many published studies, which apply different approaches, including environmental management (such as facilities design, ventilation, sprinkling, shading, *etc.*), nutritional manipulation (*i.e.*, diet formulation according to the metabolic condition of the birds), as well as inclusion of feed additives in the diet (e.g., antioxidants, vitamins, minerals, probiotics, prebiotics, essential oils, *etc.*) and water supplementation with electrolytes. Nevertheless, effectiveness of most of the interventions has been variable or inconsistent. More recently, two innovative approaches have been explored, including early-life conditioning (*i.e.*, perinatal heat acclimation) and genetic selection of breeds with increased capacity of coping with heat stress conditions (*i.e.*, increased heat tolerance). However, these potential opportunities, although promising (particularly, for poultry production in hot climatic regions), still require further research and development.

## References

[1] Selye H. (1976). Forty years of stress research: principal remaining problems and misconceptions. Can. Med. Assoc. J..

[2] Nienaber J.A., Hahn G.L. (2007). Livestock production system management responses to thermal challenges. Int. J. Biometereol..

[3] Nardone A., Ronchi B., Lacetera N., Ranieri M.S., Bernabucci U. (2010). Effects of climate changes on animal production and sustainability of livestock systems. Livestock Sci..

[4] Renaudeau D., Collin A., Yahav S., de Basilio V., Gourdine J.L., Collier R.J. (2012). Adaptation to hot climate and strategies to alleviate heat stress in livestock production. Animal.

[5] Settar P., Yalcin S., Turkmut L., Ozkan S., Cahanar A. (1999). Season by genotype interaction related to broiler growth rate and heat tolerance. Poult. Sci..

[6] Deeb N., Cahaner A. (2002). Genotype-by-environment interaction with broiler genotypes differing in growth rate. 3. Growth rate and water consumption of broiler progeny from weight-selected versus nonselected parents under normal and high ambient temperatures. Poult. Sci..

[7] Mack L.A., Felver-Gant J.N., Dennis R.L., Cheng H.W. (2013). Genetic variation alter production and behavioral responses following heat stress in 2 strains of laying hens. Poult. Sci..

[8] Mustaf S., Kahraman N.S., Firat M.Z. (2009). Intermittent partial surface wetting and its effect on body-surface temperatures and egg production of white brown domestic laying hens in Antalya (Turkey). Br. Poult. Sci..

[9] Fedde M.R. (1998). Relationship of structure and function of the avian respiratory system to disease susceptibility. Poult. Sci..

[10] Marder J., Arad Z. (1989). Panting and acid-base regulation in heat stressed birds. Comp. Biochem. Physiol. A Comp. Physiol..

[11] Elnagar S.A., Scheideler S.E., Beck M.M. (2010). Reproductive hormones, hepatic deiodinase messenger ribonucleic acid, and vasoactive intestinal polypeptide-immunoreactive cells in hypothalamus in the heat stress-induced or chemically induced hypothyroid laying hen. Poult. Sci..

[12] Ebeid T.A., Suzuki T., Sugiyama T. (2012). High temperature influences eggshell quality and calbindin-D28k localization of eggshell gland and all intestinal segments of laying hens. Poult. Sci..

[13] Donoghue D.J., Krueger B.F., Hargis B.M., Miller A.M., El Halawani M.E. (1989). Thermal stress reduces serum luteinizing hormone and bioassayable hypothalamic content of luteinizing hormone-releasing hormone in hens. Biol. Reprod..

[14] Novero R.P., Beck M.M., Gleaves E.W., Johnson A.L., Deshazer J.A. (1991). Plasma progesterone, luteinizing hormone concentrations, and granulose cell responsiveness in heat-stressed hens. Poult. Sci..

[15] Rozenboim I., Tako E., Gal-Garber O., Proudman J.A., Uni Z. (2007). The effect of heat stress on ovarian function of laying hens. Poult. Sci..

[16] Elnagar S.A., Scheideler S.E., Beck M.M. (2010). Reproductive hormones, hepatic deiodinase messenger ribonucleic acid, and vasoactive intestinal polypeptide-immunoreactive cells in hypothalamus in the heat stress-induced or chemically induced hypothyroid laying hen. Poult. Sci..

[17] Joshi P.C., Panda B., Joshi B.C. (1980). Effect of ambient temperature on semen characteristics of White Leghorn male chickens. Indian Vet. J..

[18] McDaniel C.D., Bramwell R.K., Wilson J.L., Howarth B. (1995). Fertility of male and female broiler breeders following exposure to an elevated environmental temperature. Poult. Sci..

[19] McDaniel C.D., Hood J.E., Parker H.M. (2004). An attempt at alleviating heat stress infertility in male broiler breeder chickens with dietary ascorbic acid. Int. J. Poult. Sci..

[20] Garriga C., Hunter R.R., Amat C., Planas J.M., Mitchell M.A., Moreto M. (2006). Heat stress increases apical glucose transport in the chicken jejunum. Am. J. Physiol. Reg. Integ. Comp. Physiol..

[21] Star L., Decuypere E., Parmentier H.K., Kemp B. (2008). Effect of single or combined climatic and hygienic stress in four layer lines: 2. Endocrine and oxidative stress responses. Poult. Sci..

[22] Quinteiro-Filho W.M., Ribeiro A., Ferraz-de-Paula V., Pinheiro M.L., Sakai M., As L.R., Ferreira A.J., Palermo-Neto J. (2010). Heat stress impairs performance parameters, induces intestinal injury, and decreases macrophage activity in broiler chickens. Poult. Sci..

[23] Quinteiro-Filho W.M., Gomes A.V., Pinheiro M.L., Ribeiro A., Ferraz-de-Paula V., Astolfi-Ferreira C.S., Ferreira A.J., Palermo-Neto J. (2012). Heat stress impairs performance and induces intestinal inflammation in broiler chickens infected with *Salmonella* Enteritidis. Avian Pathol..

[24] Geraert P.A., Padilha J.C., Guillaumin S. (1996). Metabolic and endocrine changes induced by chronic heat exposure in broiler chickens: biological and endocrinological variables. Br. J. Nutr..

[25] Yahav S., Hurwitz S. (1996). Induction of thermotolerance in male broiler chickens by temperature conditioning at an early age. Poult. Sci..

[26] Bobek S., Niezgoda J., Pietras M., Kacinska M., Ewy Z. (1980). The effect of acute cold and warm ambient temperatures on the thyroid hormone concentration in blood plasma, blood supply, and oxygen consumption in Japanese quail. Gen. Comp. Endocrinol..

[27] Cogburn L.A., Freeman R.M. (1987). Response surface of daily thyroid hormone rhythms in young chickens exposed to constant ambient temperature. Gen. Comp. Endocrinol..

[28] Mitchell M.A., Carlisle A.J. (1992). The effects of chronic exposure to elevated environmental temperature on intestinal morphology and nutrient absorption in the domestic fowl (*Gallus domesticus*). Comp. Biochem. Physiol. A Comp. Physiol..

[29] Boissy A., Manteuffel G., Jensen M.B., Moe R.O., Spruijt B., Keeling L.J., Winckler C., Forkman B., Dimitrov I., Langbein J., Bakken M., Veissier I., Aubert A. (2007). Assessment of positive emotions in animals to improve their welfare. Physiol. Behav..

[30] Hemsworth P.H. (2003). Human-animal interactions in livestock production. Appl. Anim. Behav. Sci..

[31] Soleimani A.F., Zulkifli I., Omar A.R., Raha A.R. (2011). Physiological responses of 3 chicken breeds to acute heat stress. Poult. Sci..

[32] Felver-Gant J.N., Mack L.A., Dennis R.L., Eicher S.D., Cheng H.W. (2012). Genetic variations alter physiological responses following heat stress in 2 strains of laying hens. Poult. Sci..

[33] Lay D.C., Fulton R.M., Hester P.Y., Karcher D.M., Kjaer J.B., Mench J.A., Mullens B.A., Newberry R.C., Nicol C.J., O’Sullivan N.P., Porter R.E. (2011). Hen welfare in different housing systems. Poult. Sci..

[34] Downing J.E.G., Miyan J.A. (2000). Neural immunoregulation: Emerging roles for nerves in immune homeostasis and disease. Immunol. Today.

[35] Padgett D.A., Glaser R. (2003). How stress influences the immune response. Trends Immunol..

[36] Butts C.L., Sternberg E.M. (2008). Neuroendocrine factors alter host defense by modulating immune function. Cel. Immunol..

[37] Marketon J.I.W., Glaser R. (2008). Stress hormones and immune function. Cel. Immunol..

[38] Ghazi S.H., Habibian M., Moeini M.M., Abdolmohammadi A.R. (2012). Effects of different levels of organic and inorganic chromium on growth performance and immunocompetence of broilers under heat stress. Biol. Trace Elem. Res..

[39] Bartlett J.R., Smith M.O. (2003). Effects of different levels of zinc on the performance and immunocompetence of broilers under heat stress. Poult. Sci..

[40] Niu Z.Y., Liu F.Z., Yan Q.L., Li W.C. (2009). Effects of different levels of vitamin E on growth performance and immune responses of broilers under heat stress. Poult. Sci..

[41] Bartlett J.R., Smith M.O. (2003). Effects of different levels of zinc on the performance and immunocompetence of broilers under heat stress. Poult. Sci..

[42] Aengwanich W. (2008). Pathological changes and the effects of ascorbic acid on lesion scores of bursa of Fabricius in broilers under chronic heat stress. Res. J. Vet. Sci..

[43] Bozkurt M., Kucukvilmaz K., Catli A.U., Cinar M., Bintas E., Coven F. (2012). Performance, egg quality, and immune response of laying hens fed diets supplemented with manna-oligosaccharide or an essential oil mixture under moderate and hot environmental conditions. Poult. Sci..

[44] Deng W., Dong X.F., Tong J.M., Zhang Q. (2012). The probiotic Bacillus licheniformis ameliorates heat stress-induced impairment of egg production, gut morphology, and intestinal mucosal immunity in laying hens. Poult. Sci..

[45] Prieto M.T., Campo J.L. (2010). Effect of heat and several additives related to stress levels on fluctuating asymmetry, heterophil:lymphocyte ratio, and tonic immobility duration in White Leghorn chicks. Poult. Sci..

[46] Droge W. (2002). Free radicals in the physiological control of cell function. Physiol. Rev..

[47] Gu X.H., Hao Y., Wang X.L. (2012). Overexpression of heat shock protein 70 and its relationship to intestine under acute heat stress in broilers: 2. Intestinal oxidative stress. Poult. Sci..

[48] St-Pierre N.R., Cobanov B., Schnitkey G. (2003). Economic losses from heat stress by US livestock industries. J. Dairy Sci..

[49] Sohail M.U., Hume M.E., Byrd J.A., Nisbet D.J., Ijaz A., Sohail A., Shabbir M.Z., Rehman H. (2012). Effect of supplementation of prebiotic mannan-oligosaccharides and probiotic mixture on growth performance of broilers subjected to chronic heat stress. Poult. Sci..

[50] Attia Y.A., Hassan R.A, Tag El-Din A.E., Abou-Shehema B.M. (2011). Effect of ascorbic acid or increasing metabolizable energy level with or without supplementation of some essential amino acids on productive and physiological traits of slow-growing chicks exposed to chronic heat stress. J. Anim. Physiol. Anim. Nutr..

[51] Imik H., Ozlu H., Gumus R., Atasever M.A., Urgar S., Atasever M. (2012). Effects of ascorbic acid and alpha-lipoic acid on performance and meat quality of broilers subjected to heat stress. Br. Poult. Sci..

[52] Estevez I. (2007). Density allowances for broilers: Where to set the limits?. Poult. Sci..

[53] Lu Q., Wen J., Zhang H. (2007). Effect of chronic heat exposure on fat deposition and meat quality in two genetic types of chicken. Poult. Sci..

[54] Dai S.F., Gao F., Xu X.L., Zhang W.H., Song S.X., Zhou G.H. (2012). Effects of dietary glutamine and gamma-aminobutyric acid on meat colour, pH, composition, and water-holding characteristic in broilers under cyclic heat stress. Br. Poult. Sci..

[55] Imik H., Atasever M.A., Urgar S., Ozlu H., Gumus R., Atasever M. (2012). Meat quality of heat stress exposed broilers and effect of protein and vitamin E. Br. Poult. Sci..

[56] Zhang Z.Y., Jia G.Q., Zuo J.J., Zhang Y., Lei J., Ren L., Feng D.Y. (2012). Effects of constant and cyclic heat stress on muscle metabolism and meat quality of broiler breast fillet and thigh meat. Poult. Sci..

[57] Mitchell M.A., Kettlewell P.J. (1998). Physiological stress and welfare of broiler chickens in transit: Solutions not problems!. Poult. Sci..

[58] Warriss P.D., Pagazaurtundua A., Brown S.N. (2005). Relationship between maximum daily temperature and mortality of broiler chickens during transport and lairage. Br. Poult. Sci..

[59] Nijdam E., Arens P., Lambooij E., Decuypere E., Stegeman J.A. (2004). Factors influencing bruises and mortality of broilers during catching, transport, and lairage. Poult. Sci..

[60] Drain M.E., Whiting T.L., Rasali D.P., D’Angiolo V.A. (2007). Warm weather transport of broiler chickens in Manitoba. I. Farm management factors associated with death loss in transit to slaughter. Can. Vet. J..

[61] Mashaly M.M., Hendricks G.L., Kalama M.A., Gehad A.E., Abbas A.O., Patterson P.H. (2004). Effect of heat stress on production parameters and immune responses of commercial laying hens. Poult. Sci..

[62] Mahmoud K.Z., Beck M.M., Scheideler S.E., Forman M.F., Anderson K.P., Kachman S.D. (1996). Acute high environmental temperature and calcium-estrogen relationship in the hen. Poult. Sci..

[63] Bonnet S., Geraert P.A., Lessire M., Carre B., Guillaumin S. (1997). Effect of high ambient temperature on feed digestibility in broilers. Poult. Sci..

[64] Zhou W.T., Fijita M., Yamamoto S., Iwasaki K., Ikawa R., Oyama H., Horikawa H. (1998). Effects of glucose in drinking water on the changes in whole blood viscosity and plasma osmolality of broiler chickens during high temperature exposure. Poult. Sci..

[65] Star L., Juul-Madsen H.R., Decuypere E., Nieuwland M.G., de Vries Reilingh G., van den Brand H., Kemp B., Parmentier H.K. (2009). Effect of early life thermal conditioning and immune challenge on thermotolerance and humoral immune competence in adult laying hens. Poult. Sci..

[66] Lin H., Mertens K., Kemps B., Govaerts T., De Ketelaere B., De Baerdemaeker J., Decuypere E., Buyse J. (2004). New approach of testing the effect of heat stress on eggshell quality: Mechanical and material properties of eggshell and membrane. Br. Poult. Sci..

[67] Farnell M.B., Moore R.W., McElroy A.P., Hargis B.M., Caldwell D.J. (2001). Effect of prolonged heat stress in single-comb white leghorn hens on progeny resistance to *Salmonella enteritidis* organ invasion. Avian Dis..

[68] Sandercock D.A., Hunter R.R., Nute G.R., Mitchell M.A., Hocking P.M. (2001). Acute heat stress-induced alterations in blood acid-base status and skeletal muscle membrane integrity in broiler chickens at two ages: Implications for meat quality. Poult. Sci..

[69] Mulder R.W.A.W. (1995). Impact of transport and related stresses on the incidence and extent of human pathogens in pig meat and poultry. J. Food Safety.

[70] Debut M., Berri C., Arnould C., Guemene D., Sante-Lhoutellier V., Sellier N., Baeza E., Jehl N., Jego Y., Beaumont C., Le Bihan-Duval E. (2005). Behavoural and physiological responses of three chicken breeds to pre-slaughter shackling and acute heat stress. Br. Poult. Sci..

[71] Dadgar S., Lee E.S., Leer T.L., Burlinguette N., Classen H.L., Crowe T.G., Shand P.J. (2010). Effect of microclimate temperature during transportation of broiler chickens on quality of the pectoralis major muscle. Poult, Sci..

[72] Gantois I., Ducatelle R., Pasmans F., Haesebrouck F., Gast R., Humphrey T.J., Van Immerseel F. (2009). Mechanisms of egg contamination by *Salmonella* Enteritidis. FEMS Microbiol. Rev..

[73] Newell D.G., Koopmans M., Verhoef L., Duizer E., Aidara-Kane A., Sprong H., Opsteegh M., Langelaar M., Threfall J., Scheutz F., van der Giessen J., Kruse H. (2010). Food-borne diseases—The challenges of 20 years ago still persist while new ones continue to emerge. Int. J. Food Microbiol..

[74] Eisenberg J.N.S., Trostle J., Sorensen R.J.D., Shields K.F. (2012). Toward a systems approach to enteric pathogen transmission: From individual independence to community interdependence. Ann. Rev. Public Health..

[75] Domingues A.R., Pires S.M., Halasa T., Hald T. (2012). Source attribution of human salmonellosis using meta-analysis of case-control studies of sporadic infections. Epidemiol. Infect..

[76] Rychlik I., Barrow P.A. (2005). Salmonella stress management and its relevance to behavious during intestinal colonization and infection. FEMS Microbiol. Rev..

[77] Humphrey T. (2006). Are happy chickens safer chickens? Poultry welfare and disease susceptibility. Br. Poult. Sci..

[78] Rostagno M.H. (2009). Can stress in farm animals increase food safety risk?. Foodborne Pathog. Dis..

[79] Verbrugghe E., Boyen F., Gaastra W., Bekhuis L., Leyman B., Van Paryz A., Haesebrouck F., Pasmans F. (2012). The complex interplay between stress and bacterial infections in animals. Vet. Microbiol..

[80] Lyte M. (2004). Microbial endocrinology and infectious disease in the 21st century. Trends Microbiol..

[81] Freestone P.P.E., Sandrini S.M., Haigh R.D., Lyte M. (2008). Microbial endocrinology: How stress influences susceptibility to infection. Trends Microbiol..

[82] Collins S.M., Surette M., Bercik P. (2012). The interplay between the intestinal microbiota and the brain. Nature Rev. Microbiol..

[83] Dinan T.G., Cryan J.F. (2012). Regulation of the stress response by the gut microbiota: Implications for psychoneuroendocrinology. Psychoneuroendocrinology.

[84] Wei S., Morrison M., Yu Z. (2013). Bacterial census of poultry intestinal microbiome. Poult. Sci..

[85] Tajima K., Nonaka I., Higuchi K., Takusari N., Kurihara M., Takenaka A., Mitsumori M., Kajikawa H., Aminov R.I. (2007). Influence of high temperature and humidity on rumen bacterial diversity in Holstein heifers. Anaerobe.

[86] Uyeno Y., Sekiguchi Y., Tajima K., Takenaka A., Kurihara M., Kamagata Y. (2010). An rRNA-based analysis for evaluating the effect of heat stress on the rumen microbial composition of Holstein heifers. Anaerobe.

[87] Burkholder K.M., Thompson K.L., Einstein M.E., Applegate T.J., Patterson J.A. (2008). Influence of stressors on normal intestinal microbiota, intestinal morphology, and susceptibility to *Salmonella* Enteritidis colonization in broilers. Poult. Sci..

[88] Traub-Dargatz J.L., Ladely S.R., Dargatz D.A., Fedorka-Cray P.J. (2006). Impact of heat stress on the fecal shedding patterns of *Salmonella enterica* Typhimurium DT104 and *Salmonella enterica* Infantis by 5-week-old male broilers. Foodborne Pathog. Dis..

[89] Patrick M.E., Christiansen L.E., Waino M., Ethelberg S., Madsen H., Wegener H.C. (2004). Effects of climate on incidence of *Campylobacter* spp. in humans and prevalence in broiler flocks in Denmark. Appl. Environ. Microbiol..

[90] Wales A., Breslin M., Carter B., Sayers R., Davies R. (2007). A longitudinal study of environmental *Salmonella* contamination in caged and free-range layer flocks. Avian Pathol..

[91] Van Der Fels-Klerx H.J., Jacobs-Reitsma W.F., Van Brakel R., Van Der Voet H., Van Asselt E.D. (2008). Prevalence of *Salmonella* in the broiler supply chain in The Netherlands. J. Food Prot..

[92] Jorgensen F., Ellis-Iversen J., Rushton S., Bull S.A., Harris S.A., Bryan S.J., Gonzalez A., Humphrey T.J. (2011). Influence of season and geography on *Campylobacter jejuni* and *C. coli* subtypes in housed broiler flocks reared in Great Britain. Appl. Environ. Microbiol..

[93] Zdragas A., Mazaraki K., Vafeas G., Giantzi V., Papadopoulos T., Ekateriniadou L. (2012). Prevalence, seasonal occurrence and antimicrobial resistance of *Salmonella* in poultry retail products in Greece. Lett. Appl. Microbiol..

